# Measurement Challenges and Solutions in Cross-National Cognitive Aging Research

**DOI:** 10.1093/geroni/igaf122.2128

**Published:** 2025-12-31

**Authors:** Alden Gross, Lindsay Kobayashi, Emma Nichols

## Abstract

Measurement precedes science and hypothesis testing. Particularly for cross-national aging research, there are profoundly important measurement issues around whether instruments measure what they are supposed to measure (validity), whether instruments provide comparable measurement across countries and contexts (invariance), and shifting reliability of measures. Presentations in this symposium highlight common challenges and propose innovative solutions to many such intractable problems with cross-national data, related to construct validation, measurement invariance, and differential measurement precision, which will be highlighted in this symposium. The first presentation by Cho and colleagues reports on a systematic review of dementia algorithms, several developed in lower and middle income countries, with a goal to provide guidance on the rubrics to evaluate dementia classification algorithms and how well they represent the construct of dementia around the world. Cantu and colleagues present an analysis of differential item functioning to uncover measurement differences between the US and Mexico in informant reports of cognitive functioning. Also speaking to the importance of evaluating measurement invariance, Gonzalez and colleagues examine measurement differences by literacy in India and Mexico, where they found differences in the measurement of language ability by literacy status in both countries. Finally, a presentation by Andrews will illustrate the value of simulation to evaluate whether differences in measurement precision of neuropsychological test batteries across country, as well as changes in precision within a country, provides biased inferences in cross-national cognitive aging research. Dr. Emma Nichols, Methods Lead for the Gateway to Global Aging Exposome Coordinating Center, will be the discussant. This is a collaborative symposium between the Brain, Epidemiology of Aging, International Comparisons of Healthy Aging, and Measurement, Statistics, and Research Design Interest Groups.

